# Inbuilt novel bioretardant feature of biopolymer isolated from *cucumis sativa* for designing drug loaded bionanosuspension

**DOI:** 10.1080/21556660.2020.1745210

**Published:** 2020-03-31

**Authors:** Yogita Tyagi, NV Satheesh Madhav

**Affiliations:** Faculty of Pharmacy, DIT University, Dehradun, India

**Keywords:** Bio-retardant, bio-nanosuspension, depression, Cucumis sativa, fluvoxamine, eudragit L-100

## Abstract

**Objectives:**

The current research work has potential for delivery of fluvoxamine moiety in bio-nanosuspension mode for the effective treatment of depression. Depression is a mood disorder characterized by persistently low mood and a feeling of sadness and loss of interest.

**Methods:**

The fluvoxamine loaded bio-nanosuspension was prepared using novel bio-retardant isolated from fruit pulp of *Cucumis sativa* by a novel method with different ratios (1:1, 1:2, 1:3, 1:4, 1:5) and the same ratios with standard polymer eudragit L-100. The bio-nanosuspensions were evaluated for pH stability studies, percentage entrapment efficacy, *in vitro* drug release, particle size, polydispersity index, zeta potential, and stability studies.

**Results:**

The bio-nanosuspension was subjected to the best formulation based on comparison of above mentioned evaluation parameters, and the Fc1 (1:1) formulation was found to be the best formulation. *Cucumis sativa* provided excellent stability for the formulation, and the resulting particle size was found to be 194 nm. The bio-nanosuspension had a Polydispersity Index (PDI) of 0.13 with zeta potential of −17.9 mV.

**Conclusion:**

The fluvoxamine loaded bio-nanosuspension using *Cucumis sativa* was found to be nontoxic and compatible with drug delivery systems for treatment of depression. This was the first report in which *Cucumis sativa* as a bioretardant demonstrated greater retardability over the standard polymer eudragit-100.

## 1. Introduction

Depression is a different from the fluctuations in mood that people experience as a part of normal life. Depression (Major depressive disorder) can be effectively treated by numerous interventions, in which antidepressant medications are most acceptable one. Electro-convulsive therapy, Psychotherapies and Cognitive behavior therapy are particularly effective for the most severe and resistant depressions^1^. Fluvoxamine is one of the antidepressants commonly used as first line treatment for major depressive disorders^2^^,^^3^ SSRIs (selective serotonin reuptake inhibitor) are antidepressant drugs that increase serotoninergic neurotransmission *via* the selective inhibition of neuronal reuptake of serotonin^4^^,^^5^. Fluvoxamine is BCS class II drug which having low solubility and high permeability but it is practically soluble in ethanol, so it is suitable for formulating nanosuspension. Nanosuspensions are carrier-free colloidal drug delivery systems that contain drug particles, stabilized by surfactant and polymers easily and prepared by suitable methods for drug delivery applications through various routes of administration. The mean sizes of the drug particles are in the nanometer range, typically between 10 and 1000 nm. Nanosized particles can increase drug solubility, the rate of dissolution and mucosal adhesion. These factors are critical in improving the bioavailability of poorly soluble drugs and in determining their effectiveness and stability. Because of their nanometer scale particle size and safe composition, nanoparticles can be delivered through various routes of administration, such as the oral, ocular and pulmonary pathways^6^. In current research work, Fluvoxamine selected as a molecule for designing a Bio-nanosuspension using novel bio-retardant and compared with standard polymer. This can be overcome by minimizing the dose as well as side-effects of API molecule used for various routes. The bio-retardant was isolated from fruit pulp of *Cucumis sativa* and characterized by IR, DSC, SEM analysis, NMR spectroscopy studies and cell-line toxicity studies. The Fluvoxamine loaded bio-nanosuspensions were developed by novel method using bio-retardant. Bio-nanosuspensions were comparatively evaluated for particle size, polydispersity index, zeta potential, pH stability studies, % entrapment efficacy, *in vitro* drug release, stability studies. In current research work, the isolation and optimization of bio-nanosuspension of Fluvoxamine is addressed along with the performance characterstics of best formulation.

## 2. Materials and methods

### 2.1. Materials

Fluvoxamine (assigned purity, 99.8%) was a gift from Life care neuro Private Limited (Baddi, Himachal Pradesh, India). *Cucumis sativa* was purchased from the market of Dehradun, Uttarakhand, India. All other chemicals and solvents were of analytical grade.

### 2.2. Isolation of bioretardant

1000 gm of fruit of *Cucumis sativa* was procured from the local market. The outer peel and seed portion was removed. The pulp was separated and ground in grinder for make a paste. The slurry was refrigerated over a period of 4 h and filtered through muslin cloth. The 300 ml of filterate was collected and treated with optimized quantity of acetone (1:2). The treated mixture was kept in refridgerator for 8 h at 4 °C to 5 °C. The biomaterial was subjected for centrifugation at 3000 rpm for a period of 15 min and collected the sediment layer. The sediment layer was dried naturally by spreading thin layer on glass plate over a period of 24 h. The dried biomaterial was purified by the hot dialysis method using an ORCHID scientific dialysis apparatus for complete removal of impurities like chlorides and sulfates. The purified bio-retardant was screened through 200#mesh and stored in descictor.The procedure was optimized by repeating six times and the percentage yield was calculated and reported^7^.

### 2.3. Characterization of bio-retardant

The novel bio-retardant was subjected to Infrared Spectroscopy (IR), Differential scanning calorimetry (DSC), Scanning electron microscopy (SEM), Nuclear magnetic resonance spectroscopy and cell-line toxicity studies.

### 2.4. Drug-excipient interaction compatibility studies

The interaction between the Fluvoxamine and Bio-retardant was determined by using the FTIR spectroscopy where infrared spectra of Fluvoxamine and bio-retardant (1:1) were taken individually first and then compared with the spectra of the combinations. The scan range was from 4000 cm^−1^ to 500 cm^−1^.

### 2.5. Preparation of bio-nanosuspensions

The Fluvoxamine loaded Bio-nanosuspensionswere prepared using novel bio-retardant isolated from fruit pulp of *Cucumis sativus* by novel method. The bio-retardant (1:1, 1:2, 1:3, 1:4, 1:5) was taken in glass mortar with drug (10 mg), 5% of dextrose and 0.9% sodium chloride, 0.1% of Poly vinyl alcohol (PVA), 0.5% benzalkonium chloride and the mixture was triturated properly for 2 mins. After that, 10 ml of distilled water was added and mixture was triturated in uniform direction in mortar pestle. The resulting solution was kept on magnetic stirrer for 30 min and then subjected for sonication for 30 min (each cycle for 3 mins) to prepared bio-nanosuspension. Similarly various formulations with different ratios were prepared by varying concentration of the bio-retardant and also prepared with standard polymer Eudragit L-100 (Table 1)^8^.

**Table 1.** Preparation formula of bio-nanosuspensions with different ratios using polymers.

Serial no.	Formula	FM1	FM2	FM3	FM4	FM5	FE1	FE2	FE3	FE4	FE5
1.	Fluvoxamine (mg)	10	10	10	10	10	10	10	10	10	10
2.	*Cucumis sativus* (bio-retardant)	0.1%	0.2%	0.3%	0.4%	0.5%	–	–	–	–	–
3.	Eudragit L-100 (Standard polymer)	–	–	–	–	–	0.1%	0.2%	0.3%	0.4%	0.5%
4.	Dextrose	5%	5%	5%	5%	5%	5%	5%	5%	5%	5%
5.	Poly vinyl alcohol (PVA)	0.1%	0.1%	0.1%	0.1%	0.1%	0.1%	0.1%	0.1%	0.1%	0.1%
6.	Sodium chloride	0.9%	0.9%	0.9%	0.9%	0.9%	0.9%	0.9%	0.9%	0.9%	0.9%
7.	Benzalkonium chloride	0.5%	0.5%	0.5%	0.5%	0.5%	0.5%	0.5%	0.5%	0.5%	0.5%
8.	Distilled water (mL)	10	10	10	10	10	10	10	10	10	10

### 2.6. Characterization of drug-loaded bio-nanosuspensions

The bio-nanosuspensions were evaluated for pH stability studies, % entrapment efficacy, *in- vitro* drug release, particle size, polydispersity index, zeta potential, stability studies.

#### 2.6.1. pH stability studies

The pH stability studies were measured at 25 °C using a caliberated pH digital meter at 20 ± 1 °C. The bio-nanosuspension was brought in contact with the electrode of pH meter and equilibrated for 1 min. This method was done in triplicate and mean was calculated along with standard deviation^9^.

#### 2.6.2. % Entrapment efficacy

The bio-nanosuspension was centrifuged at 20,000 rpm for 20 min at 5 °C temperature using cool ultracentrifuge. The amount of unincorporated drug was measured by taking the absorbance of the appropriately diluted 25 ml of supernatant solution at 268 nm using UV spectrophotometer against blank/control bio-nanosuspensions. % Entrapment efficacy was calculated by subtracting the amount of free drug in the supernatant from the initial amount of drug taken. The experiment was performed in triplicate for each batch and the average was calculated^10^. The % entrapment efficiency (EE %) could be achieved by the following equation 1:

%Entrapment efficiency=Total drug−free drug×100÷Total drug

#### 2.6.3. *In-vitro* drug release studies

The *in-vitro* drug diffusion assay was carried out in the M.S. diffusion apparatus. This was static method and requires complete replacement of the sample. The Biological membrane (egg shell membrane) was tied to the terminal portion of the cylindrical donor compartment. 2 ml of bio-nanosuspension was kept above the biological membrane in the donor compartment, and the receiver compartment was filled with diffusion medium (7.4 pH phosphate buffer). The complete sample was withdrawn at different time intervals and the receiver compartment was refilled with fresh medium. The amount of drug released was assessed by measuring the absorbance at 268 nm using UV spectrophotometer^7^.

#### 2.6.4. Particle size distribution and polydispersity index (PDI)

The average particle size and zeta potential values of the bio-nanosuspension batches were measured using a Malvern Zetasizer Nano ZS90 (Malvern Instruments) which were carried out at 25 °C using plainfolded capillary zeta cells. The diluted bio-nanosuspensions were placed directly into the cuvette and the data were collected for 10 times. All experiments were performed in triplicates and the average value was used from the each set of data^9^.

#### 2.6.5. Determination of zeta potential

PDI values were measured to understand the size distribution of the nanoparticles and the value range between 0.000 and 1.000, which demonstrates narrow to very wide size distribution of the particles^9^.

#### 2.6.6. Stability studies

Stability studies were conducted as per ICH Guidelines Q1B. Stability testing of bio-nanosuspension is done to ensure the efficacy, safety and quality of active drug substance and dosage forms and shelf life or expiration period. Stability of the bio-nanosuspensions was investigated for six months at ambient condition to monitor the change in appearance, physical characteristics and release behavior. Two portions of Fluvoxamine bio-nanosuspensions from same batch were kept under two different conditions (25ᵒ*C* ± 2 °C, 60% ± 5% RH and 40 °C ± 2 °C, 75%± 5% RH)^11^.

## 3. Results and discussions

### 3.1. Isolation of the biopolymer

The novel bio-retardant was isolated from by simplified economic process. The optimization of bio-retardant isolation process was repeated six times and the % yield was calculated. The % yield of *Cucumis sativus* was found to be of 8%w/*w* ± 2%w/w.

### 3.2. Characterization of bio-retardant

#### 3.2.1. IR spectroscopy

**Figure 1.**
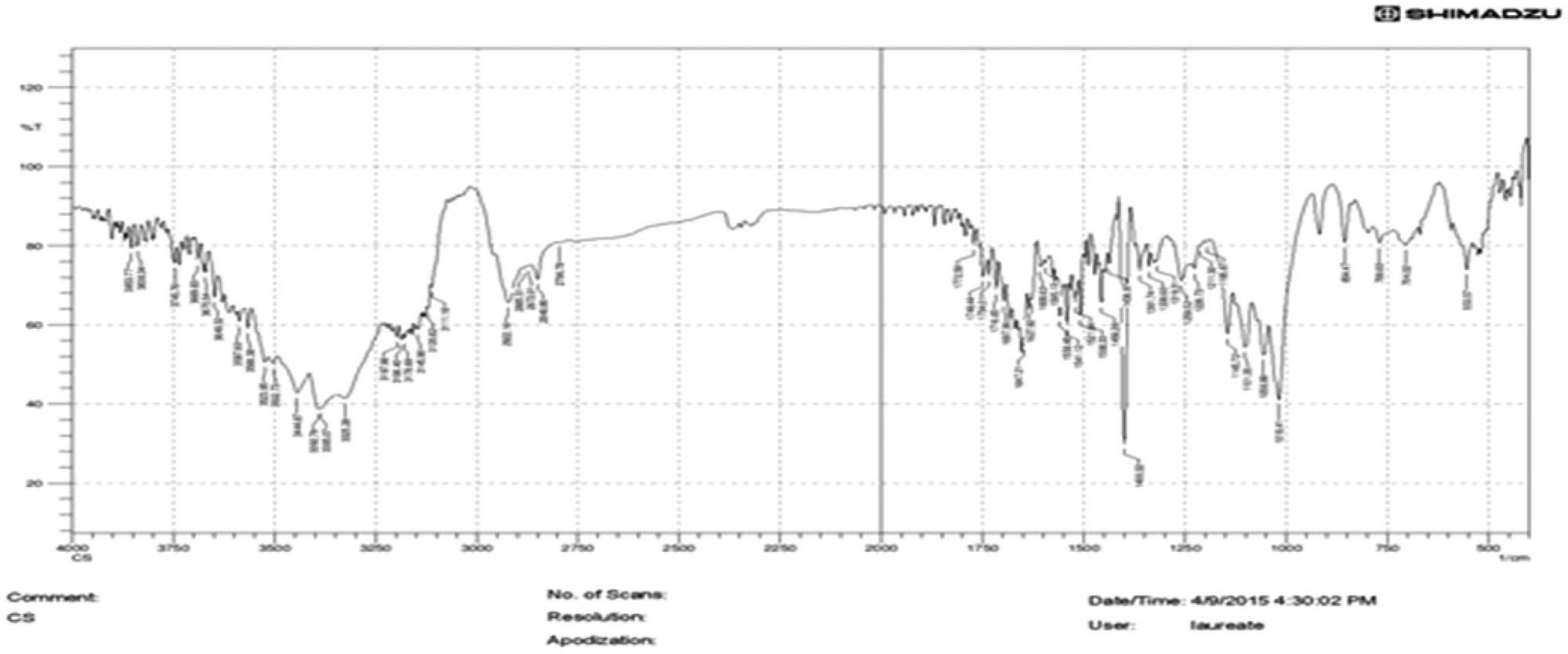
IR spectroscopy of bio-retardant *Cucumis sativus*.

The result of IR spectra of bio-retardant *Cucumis sativus* revealed the presence of Carboxlic acid RCOOH (O-H bend) at 939.52 cm^−1^, esters at 1018.32 cm^−1^, Carboxylic acid RCOO (C-O stretch) at 1400 cm^−1^, Alkenes (C = C stretch) at 1610.33 cm^−1^ and Carboxylic acid (dimer OH) at 3392.46 cm^−1^.These functional groups are responsible for retardant activity of the bio-retardant (Figure 1).

#### 3.2.2. Differential scanning calorimetry (DSC)

**Figure 2.**
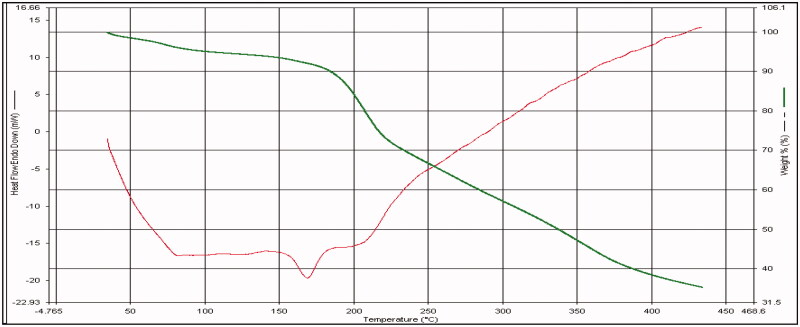
DSC of bio-retardant *Cucumis sativus*.

The DSC graph of bio-retardant *Cucumis sativus* showed melting temperature Tm is 175 °C (Figure 2).

#### 3.2.3. Scanning electron microscopy (SEM)

**Figure 3.**
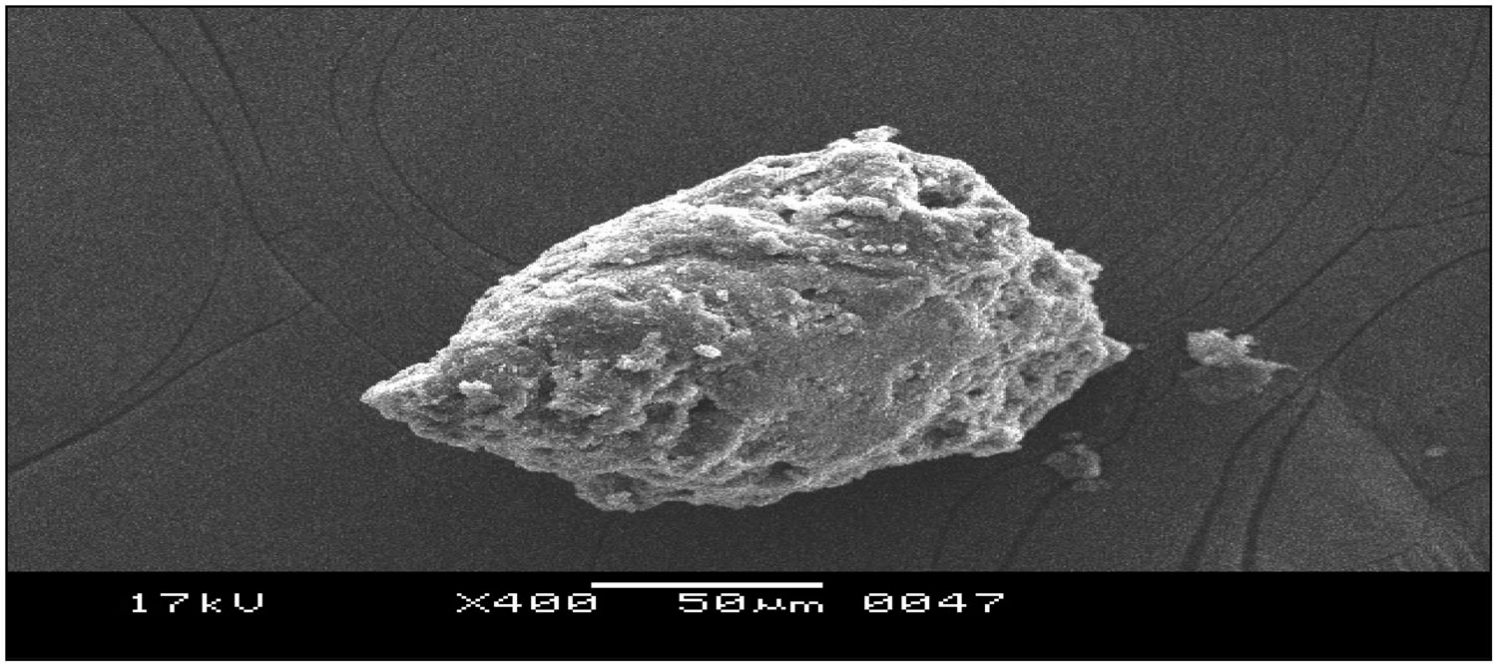
SEM of bio-retardant *Cucumis sativus*.

The surface topology of bio-retardant *Cucumis sativus* observed irregular, smooth surface with 50 μm in size at 400 magnifications. This clearly indicates that it is granular in nature and not having any type of crystal shape (Figure 3).

#### 3.2.4. Nuclear magnetic resonance (NMR)

**Figure 4.**
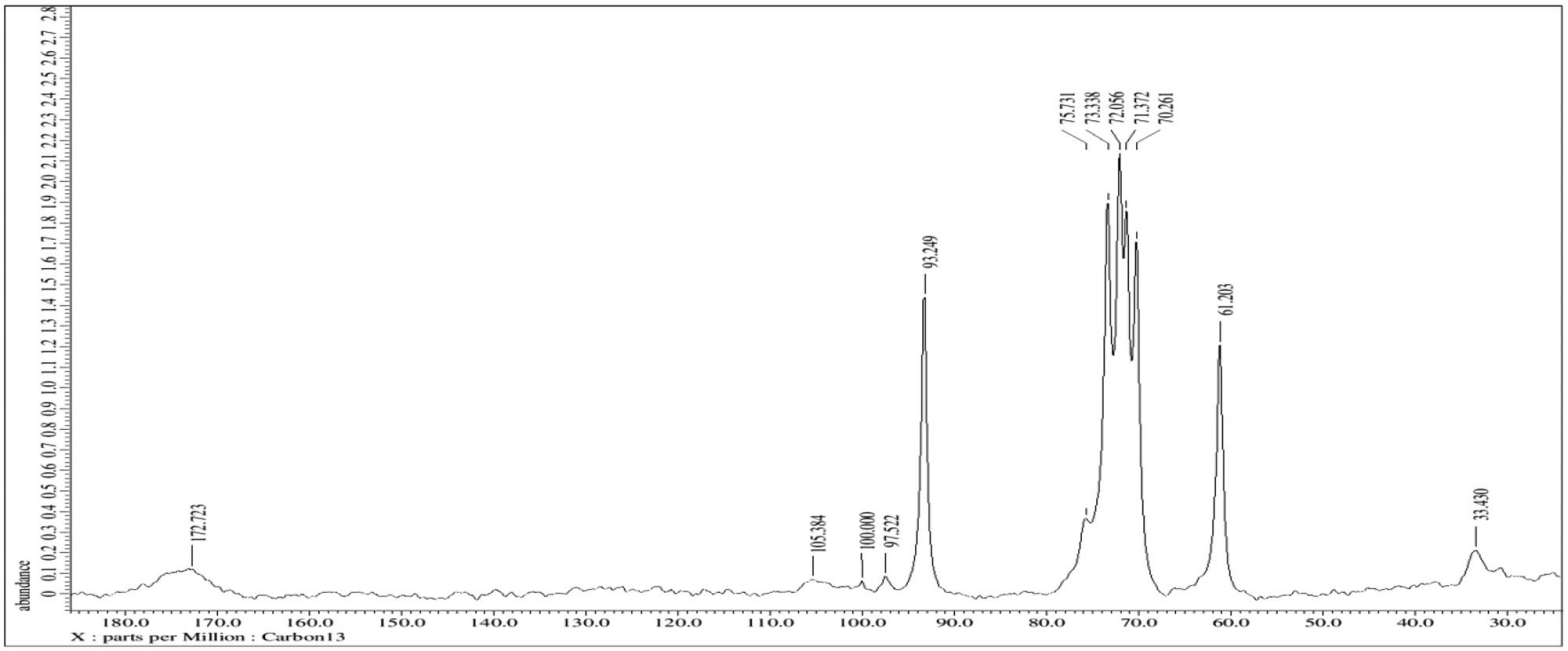
NMR of bio-retardant *Cucumis sativus*.

The NMR spectra of bio-retardant *Cucumis sativus* revealed that the peaks were found to be 33.43 ppm which showed presence of methylene carbon atom C-CH_2_, 61.20 ppm which showed presence of C-O, 72.05 ppm which showed presence of C-O, 93.24 ppm which showed presence of C = C, 172.72 ppm which showed presence of C = O preferably. Hence it clearly indicated that biomaterial was polymeric in nature (Figure 4).

#### 3.2.5. Cell-line toxicity studies

**Figure 5.**
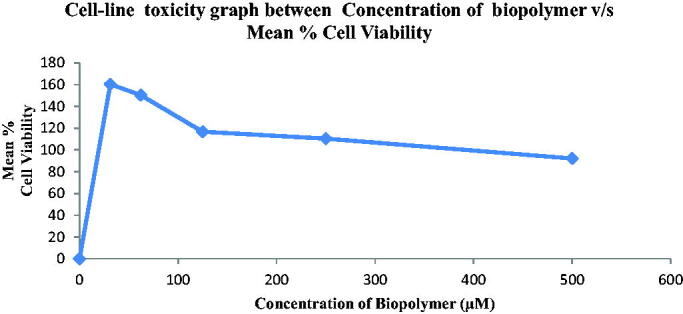
Cell-line toxicity studies of *Cucumis sativus*.

The Cell-Line toxicity for bio-retardant *Cucumis sativus* was performed by MTT Assay Method using H9c2 Cell-Line. Cell-line toxicity data of bio-retardant *Cucumis sativus* in concentrations ranging from 25–500 µM, revealed IC50 (µM) of 350.76 and mean % cell viability ranging from 160.37%−92.1%. Hence isolated bio-retardant was found to be safe and nontoxic in nature (Figure 5).

### 3.3. Drug-excipient interaction compatibility studies

**Figure 6.**
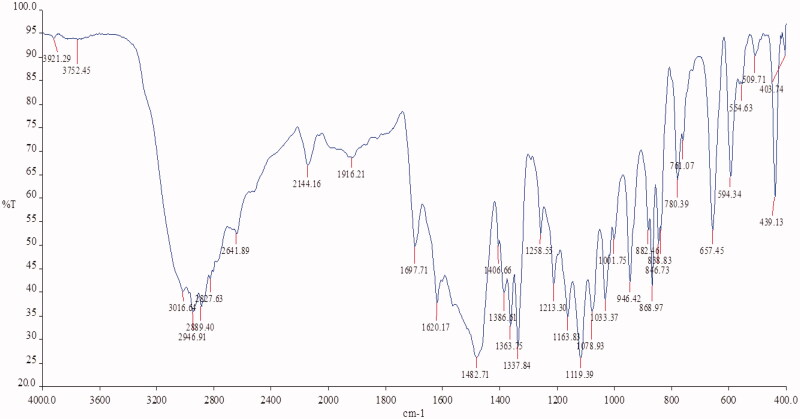
IR spectroscopy of Fluvoxamine.

**Figure 7.**
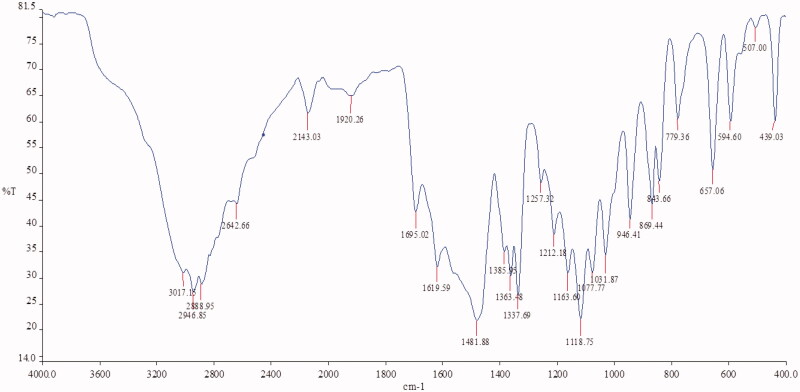
IR spectroscopy of Fluvoxamine: *Cucumis sativus*.

Drug-excipient interaction studies were performed by FTIR Spectroscopy. IR spectra of Fluvoxamine and bio-retardant showed no interaction with each other and no change in peaks of Fluvoxamine. The characteristic peaks of the Fluvoxamine (3016.64 cm^−1^, 2144.16 cm^−1^, 1697.71 cm^−1^, 1482.71 cm^−1^, 1078 cm^−1^) were appeared in the IR spectra of drug-excipient interaction (Figures 1,6,7) (Table 2).

**Table 2.** Drug-excipient compatibility studies through IR spectroscopy.

Material	Peaks (cm^−1^)	Characterstic functional group
Fluvoxamine	3016.64	C-H stretching vibration
	2144.16	C-H stretching vibration
	1697.71	C = C Stretching vibration
	1482.71	C–H Bending vibration
	1078.93	C–H Bending vibration
*Cucumis sativus*	3392.46	C–H stretching vibration
	1610.33	C = C Stretching vibration
	1400	C–H Bending vibration
	1018.32	C–H Bending vibration
	939.52	C–H Bending vibration
Fluvoxamine: *Cucumis sativus*	3016.64	C–H stretching vibration
	2144.16	C–H stretching vibration
	1697.71	C = C Stretching vibration
	1482.71	C–H Bending vibration
	1078.93	C–H Bending vibration

### 3.4. Characterization of drug-loaded bio-nanosuspensions

#### 3.4.1. Ph stability studies

**Figure 8.**
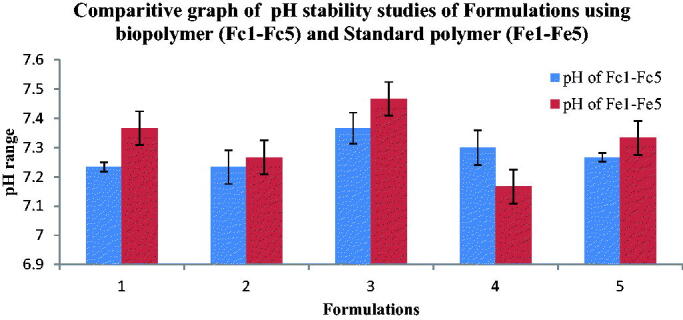
Comparitive graph of pH stability studies of Bio-nanosuspensions using bio-retardant (Fc1-Fc5) and Standard polymer (Fe1-Fe5), mean of three observation ± SD (*n* = 3).

The pH stability studies of Fluvoxamine bio-nanosuspensions prepared using bio-retardant *Cucumis sativus* (Fc1-Fc5) were found in the range of 7.2 ± 0.3 to 7.5 ± 0.2 (Figure 8) and pH stability studies of Fluvoxamine bio-nanosuspensions prepared using standard polymer (Fs1-Fs5) was found in the range of 7.2 ± 0.2 to 7.5 ± 0.4 (Figure 8).

#### 3.4.2. % Entrapment efficiency

**Figure 9.**
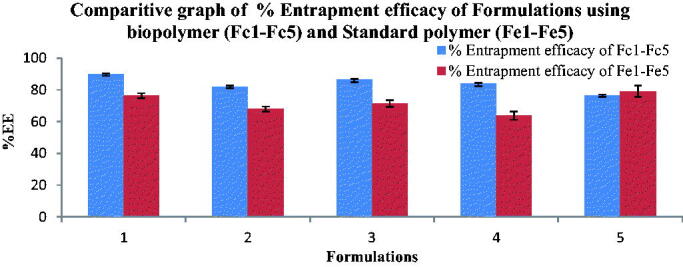
Comparitive graph of % Entrapment efficacy of Bio-nanosuspensions using bio-retardant (Fc1-Fc5) and Standard polymer (Fe1-Fe5), mean of three observation ± SD (*n* = 3).

The % Entrapment efficiency of Fluvoxamine Bio-nanosuspensions prepared using novel bio-retardant *Cucumis sativus* (Fc1-Fc5) were found in the range of 76.4% ± 4%−89.7% ± 3% (Figure 9) and % Entrapment efficacy of Fluvoxamine Bio-nanosuspensions prepared using standard polymer (Fe1-Fe5) were found in the range of 63.8% ± 2%−79.1% ± 4% (Figure 9).

#### 3.4.3. *In-vitro* drug release studies

The *In-vitro* drug release study was performed for all the Bio-nanosuspensions. The mechanism of Fluvoxamine released from the bio-nanosuspensions was studied by fitting the release data in different kinetic models such as Zero order, First order, Higuchi Matrix, Peppas Korsmeyer and Hixon Crowell and determining the *R*^2^ values of the release profile corresponding to each model. Its % drug release, T50%_%_ and T80%_%_ were calculated and based on other parameters were arranged in decreased manner.

**Figure 10.**
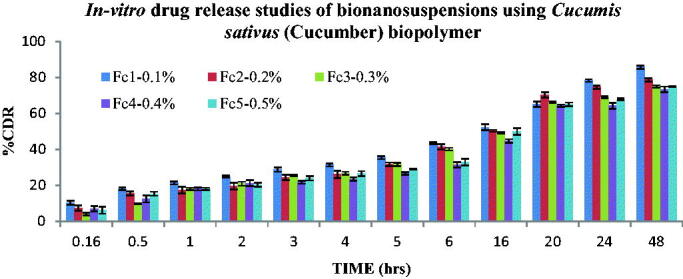
*In-vitro* drug release of bio-nanosuspensions using *Cucumis sativus* bio-retardant, mean of three observation ± SD (*n* = 3).

**Figure 11.**
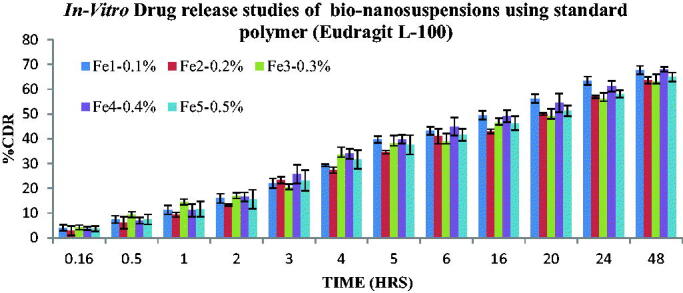
*In-vitro* drug release of bio-nanosuspensions using standard polymer (Eudragit L-100), mean of three observation ± SD (*n* = 3).

The drug release pattern for Bio-nanosuspensions Fc1-Fc5 containing bio-retardant *Cucumis sativus* based on the T50% and T80% was found to be Fc1 (0.1%) > Fc2 (0.2%) > Fc4 (0.4%)> Fc5 (0.5%) > Fc3 (0.3%). The drug release pattern for Bio-nanosuspensions Fe1-Fe5 containing standard polymer (Eudragit L-100) based on the T50% and T80% was found to be Fe4 (0.4%) > Fe2 (0.2%) > Fe3(0.3%) > Fe5(0.5%) > Fe1 (0.1%). *In-vitro* drug release was performed for all the Bio-nanosuspensions and the data indicate that Fluvoxamine loaded Bio-nanosuspensions show the sustained release behavior. Graph was plotted between %CDR and time, the *R*^2^ value, T50% and T80% was calculated from graph, from bio-retardant the Fc1 (1:1) Bio-nanosuspension was found to be the best Bio-nanosuspension showing an *R*^2^ value of 0.9814, T50% of 25.49 h and T80% of 65.25 h respectively. According to the release kinetics the best fit model was found to be Peppas Korsmeyer with Fickian Diffusion (Higuchi Matrix) as the mechanism of drug release (Figure 10 and Table 3). Where, from standard polymer (Eudragit L-100), Fe4 (1:4) Bio-nanosuspension was found to be the best formulation showing an *R*^2^ value of 0.9564, T50% of 25 h and T80% of 60 h respectively. According to the release kinetics the best fit model was found to be Peppas Korsmeyer with Fickian Diffusion (Higuchi Matrix) as the mechanism of drug release (Figure 11 and Table 3).

**Table 3.** Modeling and release kinetics of Fluvoxamine bio-nanosuspensions using bio-retardant and standard polymer.

Formulations	Zero-order *R*^2^	First-order *R*^2^	Higuchi matrix *R*^2^	Korsmeyer–Peppas equation	
				*R*^2^	*n*
Fc1	0.8193	0.9390	0.8996	0.9814	0.3707
Fc2	0.7896	0.8750	0.9106	0.9687	0.4248
Fc3	0.7773	0.8771	0.9159	0.9646	0.5022
Fc4	0.8254	0.9032	0.9056	0.9747	0.4135
Fc5	0.8111	0.8990	0.9069	0.9714	0.4227
Fe1	0.7082	0.8137	0.9319	0.9308	0.5284
Fe2	0.7201	0.8233	0.9334	0.9458	0.5650
Fe3	0.7042	0.8123	0.9248	0.9443	0.4684
Fe4	0.6553	0.8010	0.9322	0. 9564	0.4947
Fe5	0.6891	0.8103	0.9345	0. 9412	0.5339

The bio-retardant *Cucumis sativus* (Fc1-1:1) showed significant retardability with T50% of 25.49 h and T80% of 65.25 h where the data for standard polymer (Eudragit L-100) (Fs4-1:4) T50% of 25 h and T80% of 60 h. This data indicated that novel bio-retardant showed more retardability compared to standard polymer.

#### 3.4.4. Particle size distribution and polydispersity index (PDI)

**Figure 12.**
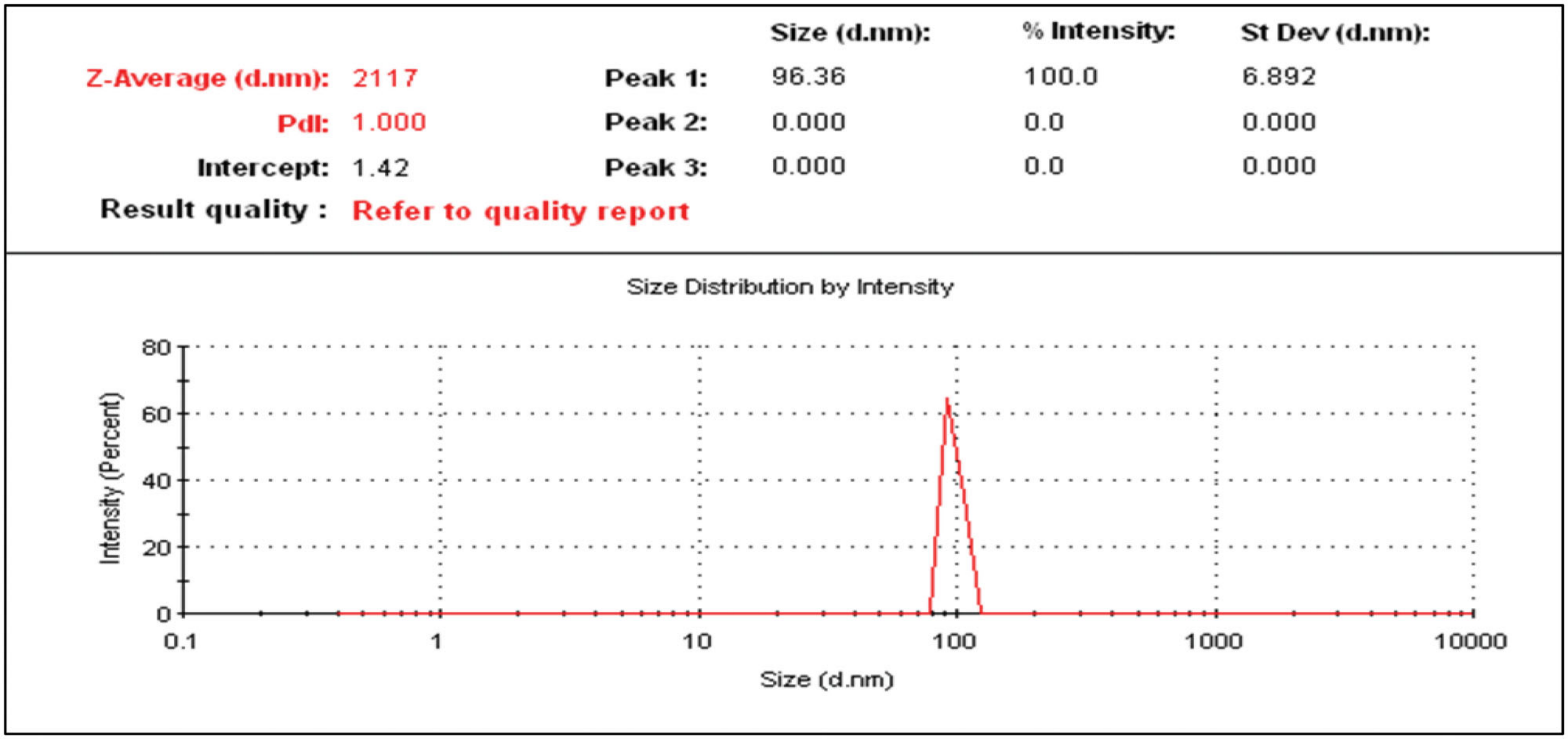
Particle size and size distribution of Fluvoxamine molecule.

**Figure 13.**
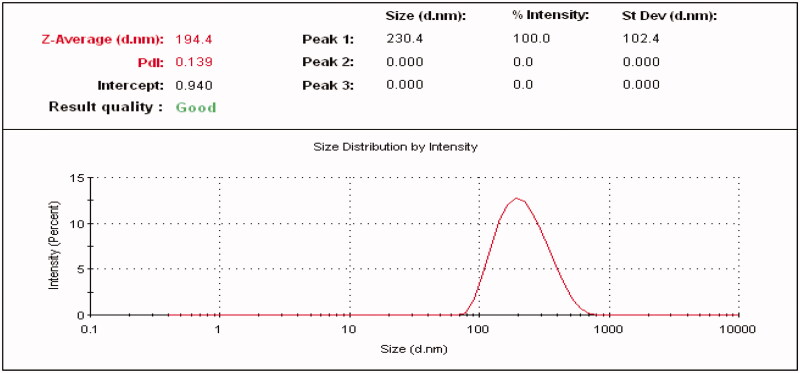
Particle size and size distribution of Fluvoxamine Bio-nanosuspension using *Cucumis sativus* (Fc1-1:1).

The z-particle size of Fluvoxamine molecule was found 2117 nm (Figure 12) and the z-particle size of Fluvoxamine loaded bio-nanosuspension (Fc1-1:1) was found 194 nm. Nanoparticles ranging 200 nm are easily captured by Kupffer cells or other phagocytic cell population that restrict bio distribution. The ability of nanoparticles to alter the biodistribution and pharmacokinetics of drug has important *in vivo* therapeutic application. So, the size and surface characteristics of nanoparticles are of prime important. These systems help in prolonging the duration of drug activity and increase the targeting efficiencies to specific site. Particle size distribution graph for formulation (Fc1) is shown in Figure 13. Polydispersity index (PDI) of Fluvoxamine molecule was found 1.0. But Polydispersity index (PDI) of Fluvoxamine bio-nanosuspension (Fc1-1:1) was found 0.13 indicating narrowest size distribution. The PDI is the measure of size distribution of the nanoparticles, where it less than 0.5 indicates monodisperse size distribution. These data also support the results observed using microscopic methods in the current study and suggest that nanosization was achieved for bio-nanosuspension.

#### 3.4.5. Determination of zeta potential

**Figure 14.**
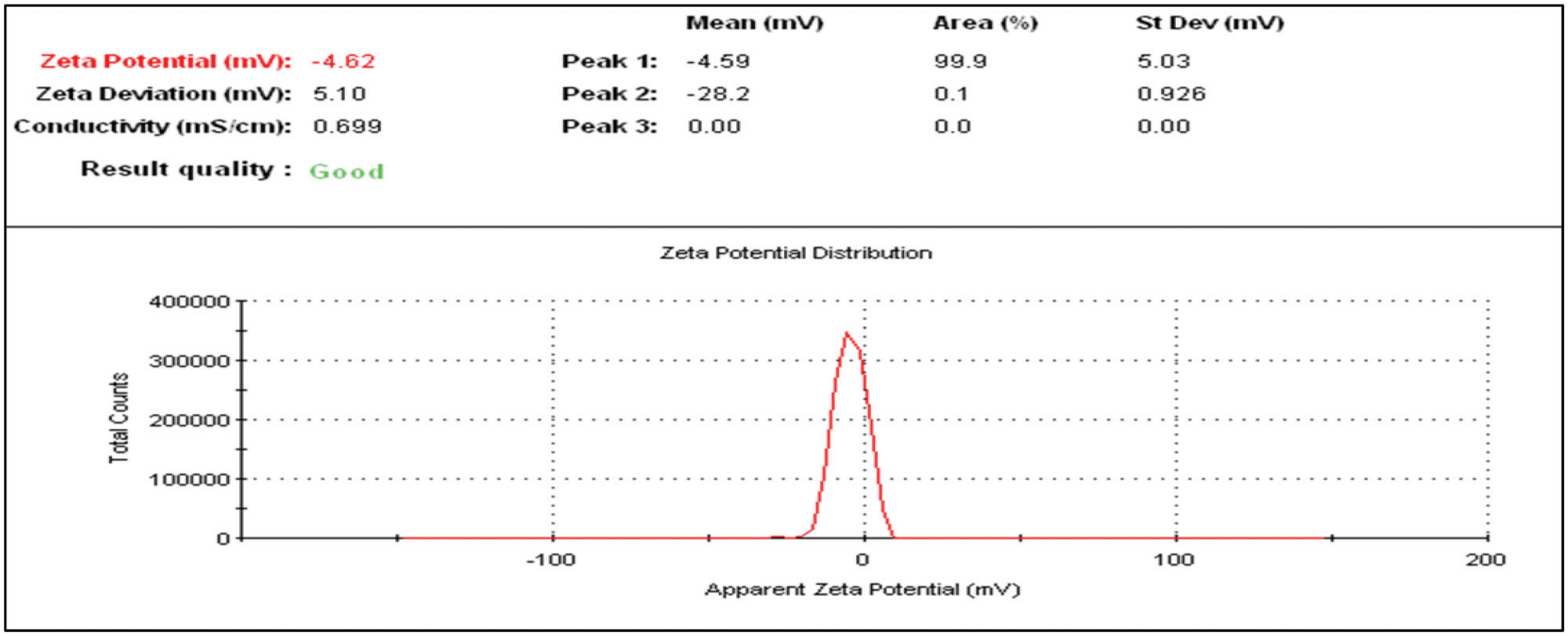
Zeta potential of Fluvoxamine molecules.

**Figure 15.**
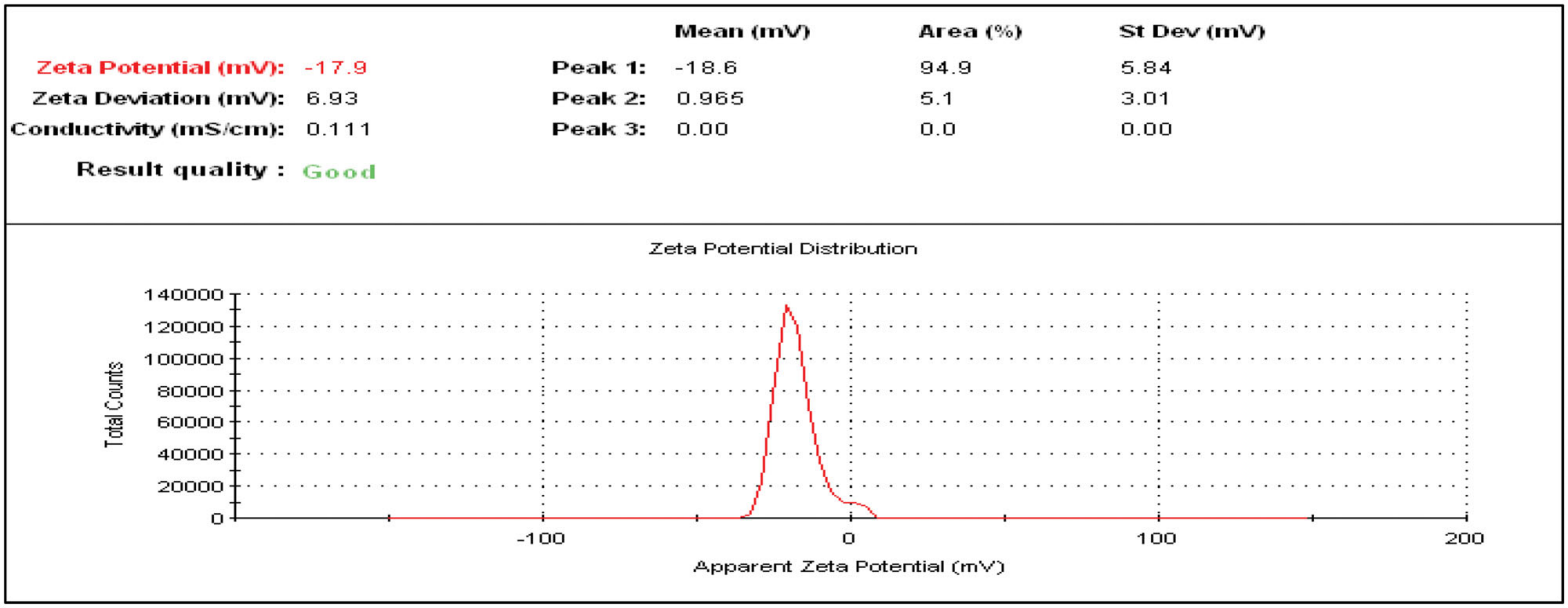
Zeta potential of Fluvoxamine bio-nanosuspension using *Cucumis sativus* (Fc1-1:1).

Fluvoxamine molecules and Fluvoxamine loaded bio-nanosuspension was evaluated by measuring the zeta potential as shown in Figures 14 and 15. Zeta potential of Fluvoxamine molecules was −4.62 mV and zeta potential of Fluvoxamine loaded bio-nanosuspension (Fc1-1:1) was −17.9 mV which indicates significant stability with no agglomeration. The value of particle surface charge indicates the stability of nanosuspensions at the macroscopic level. The zeta potential values are commonly calculated by determining the particle’s electrophoretic mobility and then converting the electrophoretic mobility to the zeta potential.

#### 3.4.6. Stability studies

**Figure 16.**
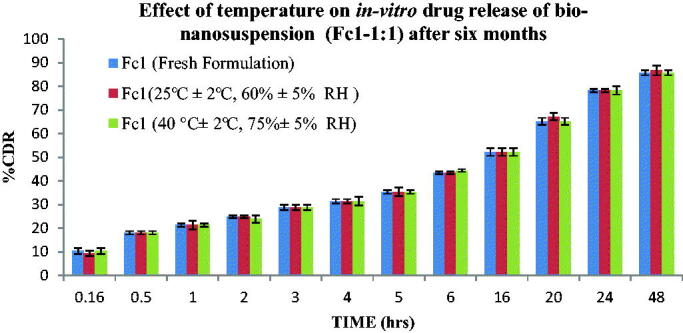
Effect of temperature on *in-vitro* drug release of bio-nanosuspension using *Cucumis sativus* (Fc1-1:1) after six months.

The Fluvoxamine bio-nanosuspension showed little to no drug loss at the end of stability studies. The Fluvoxamine loaded bio-nanosuspension using *Cucumis sativus* also showed an insignificant difference for *in-vitro* drug release. All Bio-nanosuspensions showed satisfactory drug release and other properties during and at the end of the accelerated stability period. This indicates that there was no influence on the chemical and physical stability of the Bio-nanosuspensions during the test period (Figure 16).

The isolated bio-retardant has inbuilt properties to increase the retardability of dosage form and considered as promising material for the devlopement of safe and effective drug delivery systems owing to their unique physicochemical characterstics. This bio-retardant enhances the residence time of the system and consequently the bioavailability of the Fluvoxamine.

The advantages of isolated biopolymer over the standard polymer Eudragit L-100 that the biopolymer is biodegradable and non- irritant in nature because it is isolated from edible source.This is biocompatible and nontoxic as shown in cell-line toxicity study.The advantages of isolated biopolymer *Cucumis sativus* (Fc1-1:1) showed significant retardability with T50% of 25.49 h and T80% of 65.25 h from *in-vitro* drug release graph where the data for standard polymer (Eudragit L-100) (Fs4-1:4) T50% of 25 h and T80% of 60 h from *in-vitro* drug release graph. The drug release pattern for Bio-nanosuspensions Fc1-Fc5 containing bio-retardant *Cucumis sativus* based on the T50% and T80% was found to be Fc1 (0.1%) > Fc2 (0.2%) > Fc4 (0.4%)> Fc5 (0.5%) > Fc3 (0.3%). The drug release pattern for Bio-nanosuspensions Fe1-Fe5 containing standard polymer (Eudragit L-100) based on the T50% and T80% was found to be Fe4 (0.4%) > Fe2 (0.2%) > Fe3(0.3%) > Fe5(0.5%) > Fe1 (0.1%). *In-vitro* drug release showed that the biopolymer containing bionanosuspension in concentration (Fc1-0.1%) showed best retardability than the standard polymer containing bionanosuspension in concentration (Fs4-0.4%) showed its best retardability. This data indicated that novel bio-retardant showed more retardability compared to standard polymer.

## Conclusion

The Fluvoxamine loaded bio-nanosuspensions using natural isolated bio-retardant prepared by novel method and copared with standard polymer. The isolated bio-retardant was nontoxic and bio-degradable as it extracted from the natural edible sources and compatible with drug delivery for the treatment of depression.The Fluvoxamine Bio-nanosuspensions using *Cucumis sativus* (Fc1-1:1) provided excellent stability and resulting particle size for best formulation 194 nm. The bio-nanosuspension (Fc1-1:1) had PDI of 0.13 with zeta potential of −17.9 mV which is permissible range for drug delivery. The Fluvoxamine Bio-nanosuspensions using *Cucumis sativus* is a novelistic approach significantly delivering the drug for prolonged period. The *Cucumis sativa* was served as a promising bio-retardant for delivering dosage forms and reported first time as a bioretardant which shown the retardability over the standard polymer Eudragit-100.

## Transparency

### Declaration of financial/other relationships

All authors and peer reviewers on this manuscript have no relevant financial or other relationships to disclose.
